# Transcriptome Analysis of the *Brucella abortus* BvrR/BvrS Two-Component Regulatory System

**DOI:** 10.1371/journal.pone.0010216

**Published:** 2010-04-21

**Authors:** Cristina Viadas, María C. Rodríguez, Felix J. Sangari, Jean-Pierre Gorvel, Juan M. García-Lobo, Ignacio López-Goñi

**Affiliations:** 1 Departamento de Microbiología y Parasitología, Universidad de Navarra, Pamplona, Spain; 2 Departamento de Biología Molecular, Universidad de Cantabria and Instituto de Biomedicina y Biotecnología de Cantabria (IBBTEC), UC/CSIC/IDICAN, Santander, Spain; 3 Faculté des Sciences de Luminy, Centre d'Immunologie de Marseille-Luminy (CIML), UMR6546, Aix Marseille Université, Marseille, France; 4 Inserm, U631, Marseille, France; 5 CNRS, UMR6102, Marseille, France; Charité-Universitätsmedizin Berlin, Germany

## Abstract

**Background:**

The two-component BvrR/BvrS system is essential for *Brucella abortus* virulence. It was shown previously that its dysfunction alters the expression of some major outer membrane proteins and the pattern of lipid A acylation. To determine the genes regulated by BvrR/BvrS, we performed a whole-genome microarray analysis using *B. abortus* RNA obtained from wild type and *bvrR* mutant cells grown in the same conditions.

**Methodology/Principal Findings:**

A total of 127 differentially expressed genes were found: 83 were over expressed and 44 were less expressed in the *bvrR* mutant. Two operons, the phosphotransferase system and the maltose transport system, were down-regulated. Several genes involved in cell envelope or outer membrane biogenesis were differentially expressed: genes for outer membrane proteins (*omp25a, omp25d*), lipoproteins, LPS and fatty acid biosynthesis, stress response proteins, chaperones, flagellar genes, and twelve genes encoding ABC transport systems. Ten genes related with carbon metabolism (*pckA* and *fumB* among others) were up-regulated in the *bvrR* mutant, and denitrification genes (*nirK, norC* and *nosZ*) were also regulated. Notably, seven transcriptional regulators were affected, including VjbR, ExoR and OmpR that were less expressed in the *bvrR* mutant. Finally, the expression of eleven genes which have been previously related with *Brucella* virulence was also altered.

**Conclusions/Significance:**

All these data corroborate the impact of BvrR/BvrS on cell envelope modulation, confirm that this system controls the carbon and nitrogen metabolism, and suggest a cross-talk among some regulators to adjust the *Brucella* physiology to the shift expected to occur during the transit from the extracellular to the intracellular niche.

## Introduction

Facultative intracellular bacteria such as *Brucella* must survive in varied and changing conditions ranging from the open environment to the intracellular medium. For this, the bacterium must coordinate an intricate network of factors to generate a suitable adaptive response to the various signals. This attribute is often accomplished by two-component transduction systems, consisting of a sensor kinase and a response regulator. These regulatory systems are highly conserved among bacteria and widely used for controlling gene expression in response to environmental signals. In response to stimuli, the sensor kinase autophosphorylates, then transfers its phosphate to its cognate response regulator to control the transcription of target genes [1]. Up to now, BvrR/BvrS is the best characterized two-component regulatory system of *Brucella* [2]–[4]. BvrS is a membrane-bound homodimeric protein that has three conserved regions frequently found in members of the histidine protein kinase superfamily: an amino-terminal periplasmic sensing domain with transmembrane segments, a cytoplasmic dimerisation domain with a specific His residue, and the carboxy-terminal ATP-binding kinase domain [5]. BvrR is a cytoplasmic protein that shows significant similarity to OmpR/PhoB subfamily of response regulator proteins with a specific Asp residue located within a conserved regulatory domain and an effector domain with DNA-binding activity [5].

Although genome sequencing has revealed 21 putative two-component regulatory systems in the *Brucella* genus, the best characterized one implicated in virulence is the BvrR/BvrS system. *BvrR/bvrS* mutants are avirulent in mice, have increased susceptibility to killing by nonimmune serum, show reduced invasiveness to epithelial cells and macrophages, and are incapable of inhibiting lysosome fusion and of intracellular replication [4]. As demonstrated for other two-component systems, multiple genes are expected to be under the control of BvrR/BvrS [1]. *B. abortus* mutants in this system were more susceptible to bactericidal polycationic substances like polymyxin B, melittin or poly-L-lysine, and displayed a more hydrophobic outer membrane surface than the parental strain [4]. This evidence suggests an altered outer membrane structure. Later studies demonstrated that the BvrR/BvrS system regulates transcription of at least two major outer membrane proteins, Omp22 (Omp3b) and Omp25a (Omp3a). Changes in non-protein envelope molecules such as lipid A underacylation and increased LPS acyl-chain fluidity have been also found in these mutants [3].

To further understand the role of the BvrR/BvrS two-component signal transduction system, global gene expression profiles were analyzed by using ORFeome-based *Brucella* whole-genome DNA microarrays and confirmed by reverse transcription-PCR (RT-PCR). Our results link the regulation of carbon and nitrogen metabolism to the expression of cell envelope components and suggest the existence of a complex regulatory network with the interplay of several transcriptional regulators.

## Results and Discussion

*Brucella* mutants in the BvrR/BvrS two-component regulatory system are pleiotropic [3]–[6]. Whole-genome microarray analysis was made to determine the effect of the mutation in BvrR/BvrS in the gene expression pattern of *Brucella*. *B. abortus* RNA was obtained from three independent cultures of each wild type and *bvrR* mutant cells grown in the same conditions. To confirm the reproducibility of the gene expression data, the array experiment was composed of six slides (three for each type of cells) yielding six measurements per gene, representing three biological replicates (since each gene is present twice on each slide). The ORFeome-based *Brucella* whole-genome DNA microarray used in this study has been previously validated for the analysis of gene expression under any experimental conditions [7]. The microarray experimental design was made according to the MIAME recommendations [8].

**Figure 1:**
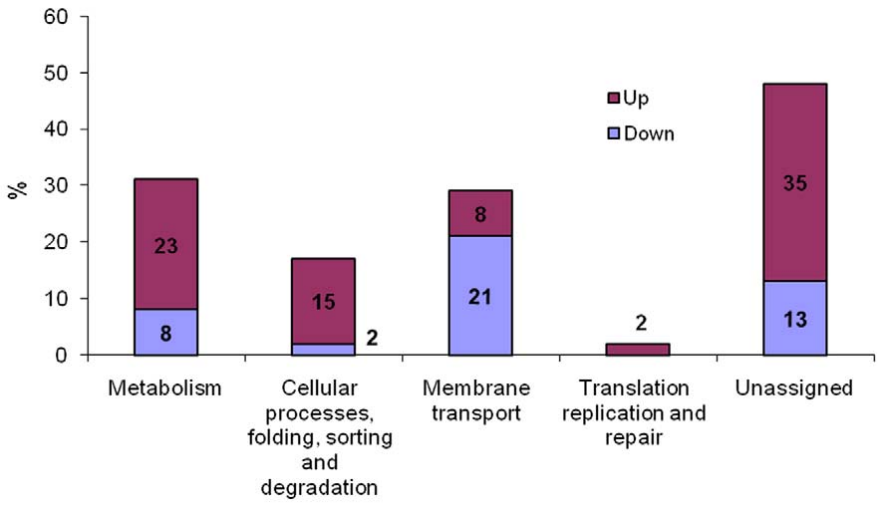
Distribution in functional categories of genes differentially expressed in *bvrR* mutant compared to parental strain. Unassigned group included eighteen genes with not pathway categories and thirty genes encoding for hypothetical proteins.

A change in gene expression was considered both statistically and biologically significant if the p-value was less than 0.01. The statistical analysis resulted in the identification of a total of 127 genes differentially transcribed in the *bvrR* mutant versus the wild type. Eighty three genes (65%) were up- and 44 (35%) were down-regulated (the complete list of differentially expressed genes in the *bvrR* mutant versus the wild type is show in Table S1). Twenty three % of the differentially transcribed genes (30) encoded for hypothetical proteins. For genes of annotated function, 59 appeared to be up regulated and 38 down regulated in the *bvrR* mutant. Genes encoding proteins involved in metabolism and cellular process are among the most up regulated genes, and those encoding proteins involved in membrane transport are among the most down regulated (Figure 1).

**Figure 2:**
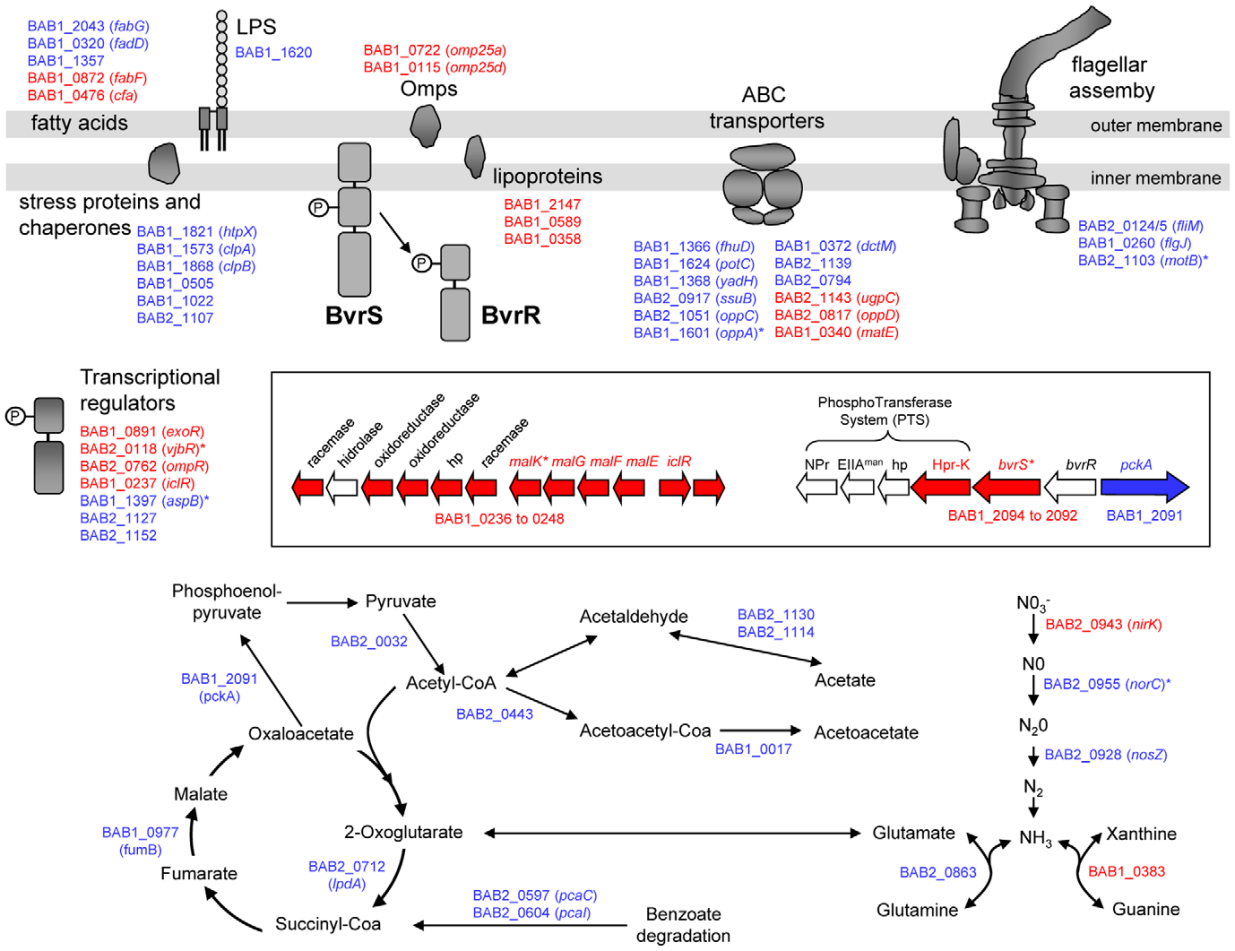
Schematic diagram of the candidate genes regulated by *Brucella* BvrR/BvrS two-component system identified in the microarray experiments. Genes up regulated are shown in blue and genes down regulated are shown in red. An asterisk shows genes that have been related previously with virulence in *Brucella*.

To further validate some data generated in the microarray experiment, forty-eight differentially expressed genes were chosen to be analyzed by real-time quantitative reverse transcription-PCR (RT-PCR). Total RNA from both *Brucella* strains were reverse transcribed into cDNA. The reactions were made by triplicate from at least two independent cultures, and the cycle of threshold (Ct) was determined for each reaction. Data were normalized by the 2^-ΔΔCt^ method [9] using the IF-1 housekeeping gene of *Brucella* as reference (Table 1). Transcriptional data of forty-one (85%) of the genes selected gave identical tendency by both methods microarray and RT-PCR: 22 were up and 19 were down regulated in the *bvrR* mutant. Interestingly, the level of transcription obtained by RT-PCR of the flagellar genes *fliM* (BAB2_0124) and *motB* (BAB2_1103), and the *pckA* gene (BAB1_2091) were the highest in the *bvrR* mutant. On the other hand, *exoR* (BAB1_0891), *omp25a* (BAB1_0722), *hpr-K* (BAB1_2094), *bvrS* (BAB1_2093) and the lipoproteins (BAB1_2147, BAB1_0589, BAB1_0358) were among the less expressed genes (Table 1). These results confirmed a good correlation between microarray and RT-PCR data, thus validating the model. Next, we will focus on the genes differentially expressed in the *bvrR* mutant (a complete representation of the differentially expressed genes is show in Figure 2).

**Table 1:** Transcriptional level of *Brucella* genes obtained by quantitative real-time PCR (RT-PCR).

*B. abortus* ORF	Gene name/Predicted function	2^-ΔΔ^Ct
BAB2_0124/5	*fliM*, flagellar motor switch protein	7.7
BAB1_2091	*pckA*, phosphoenolpyruvate carboxykinase	4.6
BAB2_1103	*motB*, flagellar motor protein	4.5
BAB2_0032	branched-chain α-keto acid dehydrogenase subunit E3	4.0
BAB2_1127	hypothetical protein	3.6
BAB2_0443	acetyl-CoA acetyltransferase	3.6
BAB1_1620	glycosyl transferase	2.6
BAB1_0017	hydroxymethylglutaryl-CoA lyase	2.4
BAB2_1130	aldehyde dehydrogenase	2.4
BAB2_0130	hypothetical protein	2.4
BAB1_0568	*iolE*, sugar phosphate isomerase/epimerase	2.1
BAB1_0320	*fadD*, acyl-CoA synthetase	2.1
BAB1_1397	*aspB*, aminotransferase	2.0
BAB1_0476	*cfa*, methyltransferase	1.9
BAB1_1366	*fhuD*, ABC transporter	1.8
BAB1_0666	*dapA*, dihydrodipicolinate synthase	1.8
BAB2_0712	*lpdA*, dihydrolipoamide dehydrogenase	1.7
BAB1_0260	*flgJ*, flagellar protein	1.6
BAB2_0863	glutaminase	1.5
BAB1_2043	*fabG*, 3-ketoacyl-(acyl-carrier-protein) reductase	1.4
BAB2_0928	*nosZ*, nitrous-oxide reductase	1.3
BAB2_0351	osmotically inducible protein C	1.3
BAB1_1624	*potC*, ABC transporter	1.2
BAB1_0977	*fumB*, fumarate hydratase	1.1
BAB1_1368	*yadH*, ABC transporter	1.0
BAB1_1821	*htpX*, heat shock protein	0.8
BAB1_0805	ATPase	0.8
BAB2_0762	*ompR*, transcriptional regulatory protein	0.8
BAB2_0943	*nirK*, nitrite reductase	0.8
BAB1_0383	guanine deaminase	0.8
BAB1_0872	*fabF*, acyltransferase	0.7
BAB1_0115	omp25d, outer membrane protein	0.6
BAB1_0246	*ucpA*, oxidoreductase	0.6
BAB1_0237	*lclR*, transcriptional regulator	0.6
BAB2_0955	*norC*, nitric-oxide reductase	0.6
BAB1_1573	*clpA*, chaperonin protein	0.6
BAB2_0118	*vjbR*, transcriptional regulator	0.5
BAB1_0239	*malF*, maltose transporter permease sugar	0.4
BAB2_1152	transcriptional regulator, AraC family	0.4
BAB1_0358	lipoprotein	0.3
BAB1_2094	*hpr-K*, phospho transferase system	0.2
BAB1_2093	*bvrS*, sensor protein	0.2
BAB1_2147	lipoprotein	0.1
BAB1_0716	glycoprotein	0.1
BAB1_0526	polysaccharide deacetylase	0.1
BAB1_0589	lipoprotein	0.1
BAB1_0891	*exoR*, exopolysacchride production negative regulator	0.0
BAB1_0722	*omp25a*, outer membrane protein	0.0

The results are expressed as 2^-ΔΔCt^ . Figures  = 1 indicate that the gene is expressed similarly in both conditions (*bvrR* mutants versus the wild type strain), figures >1 indicate that the gen is over expressed in the *bvrR* mutant, and figures <1 indicate that the gen is less expressed in the mutant.

### Cell envelope modulation

It is well know that the transcription of *omp25a* y *omp22* genes is under the control of the BvrR/BvrS system, and that *bvrR/bvrS* mutants have increased amount of underacylated lipid A species in the LPS [2], [3]. In addition, proteomic analysis of *Brucella* outer membrane fragments demonstrated that the expression of several OMPs, lipoproteins and chaperones was altered in these mutants [6]. These observations led to the hypothesis that the BvrR/BvrS system is involved in cell envelope changes required for adaptation to the intracellular environment. Our microarray results demonstrated several genes directly involved in cell envelope or outer membrane biogenesis differentially expressed in the *bvrR* mutant. As expected, these included genes that encoded OMPs like Omp25a (BAB1_0722) and Omp25d (BAB1_0115) which were down-regulated. Other *bvrR* regulated genes related with cell envelope were: three lipoprotein genes (BAB1_0358; BAB1_0589; BAB1_2147), which were down-regulated; six genes for periplasmic proteins and chaperones (*htpX*, heat shock protein, BAB1_1821; *clpA* and *clpB*, stress response proteins, BAB1_1573 and BAB1_1868, respectively; BAB2_1107; BAB1_0505; BAB1_1022), which were all up-regulated; one gene related with LPS biosynthesis (glycosyl transferase, BAB1_1620), which was up-regulated; and five genes for fatty acids biosynthesis (*fabG*, ketoacyl-acyl-carrier-protein reductase, BAB1_2043; *fabF*, oxoacyl-acyl-carrier-protein synthase, BAB1_0872; *fadD*, fatty-acyl-CoA synthase, BAB1_0320; *cfa*, cyclopropane-fatty-acyl-phospholipid synthase, BAB1_0476; BAB1_1357). These data confirm that BvrR/BvrS regulates bacterial envelope changes that could modify surface properties relevant for *Brucella* virulence [6].

### Regulation of carbon and nitrogen metabolism

One of the remarkable findings observed in our microarray analysis was that several genes related with metabolism were also differentially expressed. These included twelve genes encoding ABC transport systems (*matE*, BAB1_0340; *dctM*, BAB1_0372; *fhuD*, BAB1_1366; *yadH*, BAB1_1368; *oppA*, BAB1_1601; *oppC*, BAB2_1051; *oppD*, BAB2_0817; *potC*, BAB1_ 1624; *ssuB*, BAB2_0917; *ugpC*, BAB2_1143; BAB2_0794; BAB2_1139), ten genes related with carbohydrate, amino or fatty acids metabolism and five related with nitrogen metabolism. Interestingly, all genes related with carbohydrate, amino or fatty acids metabolism were up-regulated in the *bvrR* mutant. These included the first enzyme in gluconeogenesis (*pckA*, phosphoenolpyruvate carboxykinase, BAB1_2091), four genes involved in TCA cycle and pyruvate metabolism (*fumB*, fumarate hydratase, BAB1_0977; *lpdA*, dihydrolipoamide dehydrogenase, BAB2_0712; pyruvate dehydrogenase, BAB2_0032; acetyl-CoA acetyltransferase, BAB2_0443), three genes involved in amino or fatty acid metabolism (aldehyde dehydrogenases, BAB2_1130, BAB2_1114; hydroxymethylglutaryl-CoA lyase, BAB1_0017), and two genes involve in benzoate degradation (*pcaC*, carboxymuconolactone decarboxylase, BAB2_0597; *pcaI*, coenzyme A transferase, BAB2_0604). In addition, the complete maltose transport system of *Brucella*, which consists in a large operon containing thirteen genes (BAB1_0236-0248) was also affected. Ten of these genes, including *malK*, *malG*, *malF*, *malE* and an *iclR* regulator, were down-regulated suggesting that the complete operon was negatively regulated in the *bvrR* mutant.

**Figure 3:**
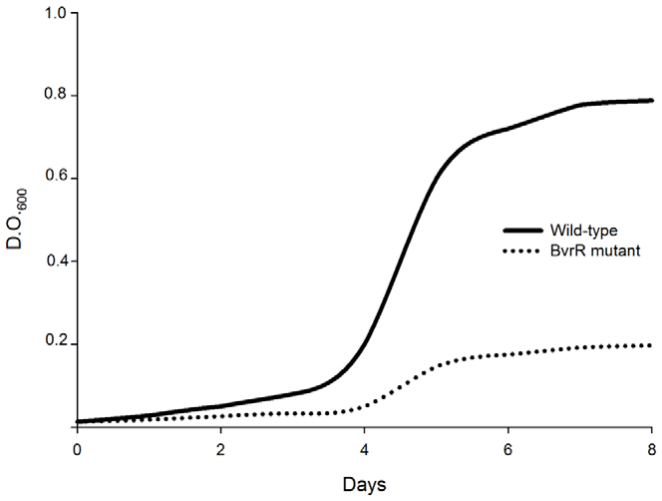
Growth curves of the parental *B. abortus* 2308 and the *bvrR* mutant in synthetic minimal media.

Although it has been showed that the mutants in the BvrR/BvrS system have no obvious defects with regard to the ability to grow on standard media [4], our microarray results suggests that the BvrR/BvrS system controls elements directly involved in adjusting the *Brucella* metabolism to the nutrient shift expected to occur during the transit to the intracellular niche. To determine if the BvrR/BvrS system affects the metabolism, *bvrR* mutant and wild type strains were grown in synthetic minimal media. As show in Figure 3, growth of the *bvrR* mutant was significantly reduced in minimal media.

Other genes differentially expressed in the *bvrR* mutant included denitrification genes. The nitrite reductase gene (*nirK*, BAB2_0943) was down regulated and the nitric oxide and nitrous oxide reductases genes (*norC*, BAB2_0955; *nosZ*, BAB2_0928) were up regulated. On the other hand, two deaminases (glutaminase, BAB2_0863; guanine deaminase, BAB1_0383) were also affected. Since *Brucella* is an intracellular facultative pathogen, the bacteria could use these denitrification reactions to grow under low-oxygen condition by respiration of nitrate. *Brucella* may also take advantage of denitrification to cope with nitric oxide (NO) production in the macrophage during the innate response against infection. In fact, some of these denitrification genes have been related with the virulence in mice [10], [11]. Interestingly, our experiments to study the intracellular transcriptional level of BvrR/BvrS controlled genes (see below) showed that whereas *norC* was induced intracellularly, *nirK* and *nosZ* were less expressed. Taken together all these data support the proposal that one role of the BvrR/BvrS system could be neutralize the production of toxic reactive nitrogen molecules, as NO, by the host. These results also demonstrated a connection between carbon and nitrogen metabolism and BvrR/BvrS in *Brucella*.

Our results also demonstrated that gene *hpr-K* (BAB1_2094), a member of the *Brucella* phosphotransferase system (PTS; BAB1_2097-2094) adjacent to the *bvrR/bvrS*, was down-regulated. As mentioned before, phosphoenolpyruvate carboxykinase gene (*pckA*, BAB1_2091) which is located upstream of the regulatory gene *bvrR* and divergently expressed was up-regulated. Comparative genome analysis revealed that in addition to the *bvrR/bvrS* genes, the genome structure around these genes is essentially the same for all the α-proteobacteria [5]. Genes encoding proteins related to the PTS, including a HPr Ser-kinase, an EIIA permease of the mannose family and a HPr homologue precede those of the two-component regulatory system. In most of these loci, upstream of the regulatory gene the *pckA* is divergently expressed (Figure 2). This gene catalyzes the reversible decarboxylation and phosphorylation of oxaloacetate to form phosphoenolpyruvate. In α-proteobacteria, it has been proposed that HPr might control the phosphorylation state of the transcription regulator [12]–[14]. In this regard, Letesson and col. [15] have suggested that in *Brucella* the PTS could interact with the BvrS sensor kinase, which in turn phosphorylates the response regulator. Then, the BvrR could control transcription of the *pckA* gene, which encodes an essential control enzyme of the gluconeogenesis and Krebs cycle. This hypothesis could explain the observation that mutants in the regulatory gene *bvrR* were inhibited in minimal media (see above). According to this, it has been demonstrated that in *A. tumefaciens* the *pckA* genes is indeed under the control of ChvG/ChvI [16] and that null mutants in *S. meliloti exoS* and *chvI* have pleiotropic growth defects and were unable to grow on several carbon sources [17]. A link between carbon and nitrogen metabolism, PTS and two-component regulatory systems have been proposed for some bacteria [18], and our microarray results strongly suggest that same relationship could be made for *Brucella*.

### BvrR/BvrS and the expression of other transcriptional regulators

Notably, seven transcriptional regulators genes were also differentially expressed in the *bvrR* mutant compared to parental strain: BAB1_0237, BAB1_0891 (*exoR*), BAB1_1397, BAB2_0118 (*vjbR*), BAB2_0762 (*ompR*), BAB2_1127 and BAB2_1152. Three of these, namely *exoR, ompR* and *vjbR* are down regulated and have been previously implicated in *Brucella* virulence. *B. melitensis vjbR* mutant is highly attenuated in both cellular and mouse models of infection [19]. VjbR has been described as a transcriptional regulator able to activate directly the secretion system *virB* operon and the flagellar genes, both virulence factors associated to the surface of the bacteria [20], [21]. Moreover, it has been demonstrated that VjbR controls the synthesis of exopolysaccharides and the productions of several OMPs, some of which are also involved in virulence [22]. Interestingly, our results also showed that the expression of *vjbR* was also induced intracellularly (see below). All these data suggest that VjbR, similarly to the BvrR/BvrS system, is involved in the control of outer membrane composition and virulence. In addition, DeJong and col [20] have demonstrated that among the promoters which expression is dependent of the VjbR regulator, is the *ompR* gene, the regulator of the OmpR/EnvZ two component system. *E. coli* OmpR/EnvZ system controls the transcription of the outer membrane porins OmpF and OmpC in response to osmolarity [23]. Moreover, a systematically transcriptome analysis of all two component regulatory systems in *E. coli* has demonstrated that the OmpR/EnvZ system also controls the metabolism of amino acids, flagellar synthesis and nutrient transport [24]. As we have show in this study, the expression of at least three flagellar genes (*fliM*, BAB2_0124/5; *flgJ*, BAB1_0260; *motB*, BAB2_1103) were increased also in the *bvrR* mutant (Figure 2).

The two-regulatory systems ChvG(ExoS)/ChvI in *S. meliloti* and *A. tumefaciens* posses a high level of identity with the *Brucella* BvrR/BvrS [25], [26]. Chaves-Olarte et al have reported that *B. abortus bvrS* mutant complemented with the ExoS protein recuperated the ability to invade and replicated successfully in HeLa and macrophage cells [27], suggesting that the BvrR/BvrS system is functionally interchangeable with the ExoS/ChvI system. *A. tumefaciens* ChvI/ChvG system controls the expression of the Aop, an OMP homologous to *Brucella* Omp25a [28], and in *S. meliloti*, ExoS/ChvI is a key regulator of gene expression for exopolysaccharide synthesis, motility and nutrient utilization [26]. It has been described in *S. meliloti* that *exoR* gene encodes a global regulator of transcription and that ExoR interacts genetically with both ExoS and ChvI and inhibits ExoS/ChvI activity [29], [30]. Further analysis indicated that both the ExoR protein and the ExoS/ChvI two-component regulatory system are involved in the regulation of both polysaccharides and flagellum biosynthesis [31]. In addition, the transcription of the *S. meliloti lpsS* gene, that encodes a sulfotransferase that modifies LPS, is dependent on the *exoR* gene [32]. Other authors [29] suggest that ExoR is an inhibitor of two-component signaling that may be conserved in a large number of α-proteobacteria. Our results also support this hypothesis: the functional relationship between the *exoR* gene and the BvrR/BvrS system. Based on all these findings, obvious comparison about the function of all these regulators in *Brucella* could be made. The fact that the expression of VjbR, OmpR and ExoR was altered in the *bvrR* mutant demonstrated for the first time an interaction or cross-talk among these global regulators, all involved in the control of composition and structure of the cell envelope (OMPs, LPS, chaperones, flagella, …).

### Virulence and the adaptation to intracellular growth

*BvrR/bvrS* mutants are unable to multiply intracellularly and are avirulent in the mouse model [4]. Our microarray results demonstrated that at least 127 genes were differentially expressed in the *bvrR* mutant. Although this general expression changes could explain the complete loss of virulence of these mutants, it was remarkable the presence among them of ten genes, in addition to *bvrS*, whose products are already known to be associated with *Brucella* virulence [10], [11], [33], [34]. These included the already mentioned *vjbR*, but also *motB* (BAB2_1103), *malK* (BAB1_0241), *norC* (BAB2_0955), *oppA* (BAB1_1601), *aspB* (BAB1_1397), *mosA* (BAB1_0666) and three genes encoding hypothetical proteins (BAB1_1717, BAB1_0597 y BAB2_1127). *B. melitensis malK* mutant and *B. suis aspB* mutant were attenuated in cellular model of infection, and *B. melitensis* mutants in *vjbR, motB, oppA, mosA* and the hypothetical proteins BAB1_0597, BAB1_1717, BAB2_1127 were attenuated in both cellular and mouse models of infection (for a review see [33], [34]). In addition, it has been demonstrated that some denitrification genes of the *nor* operon are required for *Brucella* virulence: *norD* in *B. suis* and *norB* in *B. melitensis* [10], [11].

Most of the genes candidate to be regulated by BvrR/BvrS identified in our microarray experiments can be involved with the changes needed for intracellular survival of *Brucella*. In order to investigate if the BvrR/BvrS controlled genes were expressed intracellularly, bacterial RNA was obtained from *B. abortus* wild type recovered from infected cells as described in Material and Methods. The amount of bacterial RNA was not enough to perform microarray hybridizations. For this reason, the analysis of intracellular expression of 32 selected genes was done by RT-PCR by using total RNA from intracellular bacteria and from the same strain (*B. abortus* 2308) grown in laboratory conditions (Table 2). *VirB8* (BAB2_0061) was used as control of intracellularly expressed gene [35]. The results showed significant differences in the expression of at least fifteen genes controlled by BvrR/BvrS. The expression of genes *vjbR*, *malF, norC, pckA, fumB*, BAB1_0017 (fatty acids metabolism) and BAB1_1620 (LPS glycosyl transferase) were induced intracellularly. On the other hand, two genes for cell envelope proteins (*omp25d* and one lipoprotein) and three denitrification genes (*norC*, *nirK* and glutaminase BAB2_0863) were less expressed intracellularly.

**Table 2:** Intracellular transcriptional level of *B. abortus 2308* genes candidates to be controlled by BvrR/BvrS.

*B. abortus* ORF	Gene name/Predicted function	2^-ΔΔ^Ct
BAB2_0061	*virB8*, type IV secretion system	39.5
BAB1_0239	*malF*, maltose ABC transporter permease sugar	2.9
BAB2_0955	*norC*, nitric-oxide reductase	2.5
BAB1_0017	hydroxymethylglutaryl-CoA lyase	2.3
BAB2_0118	*vjbR*, transcriptional regulator	2.0
BAB1_1620	glycosyl transferase	1.9
BAB1_2091	*pckA*, phosphoenolpyruvate carboxykinase	1.6
BAB1_0977	*fumB*, fumarate hydratase	1.5
BAB2_0762	*ompR*, transcriptional regulatory protein	1.3
BAB1_0476	*cfa*, methyltransferase	1.3
BAB1_2043	*fabG*, 3-ketoacyl-(acyl-carrier-protein) reductase	1.2
BAB1_0237	*IclR*, transcriptional regulator	1.1
BAB1_0320	*fadD*, acyl-CoA synthetase	1.0
BAB1_0260	*flgJ*, flagellar protein	1.0
BAB1_0891	*exoR*, exopolysacchride production negative regulator	1.0
BAB1_0872	*fabF*, acyltransferase	0.9
BAB1_0246	*ucpA*, oxidoreductase	0.8
BAB1_0358	Lipoprotein	0.8
BAB2_0443	acetyl-CoA acetyltransferase	0.8
BAB2_1130	aldehyde dehydrogenase	0.8
BAB1_0383	guanine deaminase	0.7
BAB1_2094	*hpr-K*, phospho transferase system	0.7
BAB1_0722	*omp25a*, outer membrane protein	0.7
BAB1_2093	*bvrS*, sensor protein	0.6
BAB1_2147	Lipoprotein	0.6
BAB1_2092	*bvrR*, DNA-binding response regulator	0.5
BAB2_0928	*nosZ*, nitrous-oxide reductase	0.5
BAB1_0589	Lipoprotein	0.4
BAB2_0032	branched-chain α-keto acid dehydrogenase subunit E3	0.4
BAB1_0115	omp25d, outer membrane protein	0.3
BAB2_0863	glutaminase	0.3
BAB2_0943	*nirK*, nitrite reductase	0.3

The results are expressed as 2^-ΔΔCt^ . Figures  = 1 indicate that the gene is expressed similarly in both conditions (*bvrR* mutants versus the wild type strain), figures >1 indicate that the gen is over expressed in the *bvrR* mutant, and figures <1 indicate that the gen is less expressed in the mutant.

In conclusion, all these results and previous findings support the proposal that BvrR/BvrS controls a significantly broad set of phenotypes and define an important and coordinate gateway between the free-living and intracellular states of *Brucella*. However, 30 of the genes differentially expressed in the *bvrR* mutant compared with the parental strain have a yet uncharacterized function. This group may contain unknown essential information to completely understand the regulatory role of the BvrR/BvrS two-component regulatory system.

## Materials and Methods

### Bacterial strains and growth conditions

Bacterial strains used in the present study were *B. abortus* 2308 (parental, wild type, virulent strain) and *B. abortus* 65.21 (*bvrR*:Tn5 mutant, avirulent) [4]. Cells were grown in 10 mL of Tryptic Soy Broth (TSB; Biomerieux; Trypticase 17 g/L, Soyase 3 g/L, NaCl 5 g/L, K_2_PO_4_ 2.5 g/L, glucose 2.5 g/L, final pH = 7.3) into a 100-mL flask on an orbital shaker (200 rpm) at 37°C until mid log phase (OD_600_ = 0.6–0.7). Alternatively, cells were grown in a modification of the *Brucella* synthetic liquid medium of Gerhardt [36] supplemented with glucose (glucose 1 g/L, lactic acid 5.9 g/L, glycerol 30 g/L, glutamate 5 g/L, thiamine-HCl 0.2 mg/L, nicotinic acid 0.2 mg/L, pantothenic acid 0.04 mg/L, biotin 0.0001 mg/L, K_2_HPO_4_ 10 g/L, Na_2_S_2_O_3_ 5H_2_O 0.1 g/L, SO_4_Mg_2_ 10 mg/L, SO_4_Mn_2_ 0.1 mg/L, SO_4_Fe_2_ 0.1 mg/L, NaCl 7.5 g/L, final pH = 7.0).

### Isolation of RNA from *Brucella*

The *Brucella* RNA for microarray analysis was purified and amplified by the MessageAmp II-Bacteria RNA Amplification Kit (Ambion), which enables prokaryotic RNA amplification for whole genome expression analysis from bacterial samples. Briefly, the bacterial cell culture was stabilized with the Protect Bacteria Reagent (Ambion), and total RNA was extracted with the RNeasy Mini System (Qiagen) in combination with the RNase-Free DNase Set (Qiagen) according to the manufacturer's instructions. *Brucella* mRNA was enriched using MICROBExpress Kit (Ambion), and antisense amino-allyl dUTP marked RNA (aRNA) was obtained by amplification with the MessageAmp II-Bacteria kit (Ambion), following the manufacturer's instructions. RNA preparations were tested for the lack of *Brucella* genomic DNA contamination by PCR with primers specific for the IF-1 *Brucella* gene. The absence of residual DNA of BHK-21 cells (see below) was confirmed by the lack of a product after PCR with primers specific for actin eukaryotic gene [37]. Concentration of RNA was determined using the NanoDrop ND-1000. Samples were stored at −80°C until used.

### Construction of *Brucella* DNA microarray

A whole-genome DNA microarray based on the PCR products of predicted ORFs from the *B. abortus* genome was used for global gene expression analysis. Details of the construction and evaluation of the microarray were described previously [7]. Briefly, each *Brucella* ORF was amplified by PCR from the complete *Brucella* ORFeome library [38] and PCR products were purified using the Montage PCRμ96 Cleanup System (Millipore), dried, resuspended in 50% dimethylsulfoxide (v/v), and arrayed into 384-well plates. PCR products were printed in duplicated onto UltraGAPs Coated Slides (Corning Life Sciences) using MicroGrid II 610 Robotic System (Genomic Solutions). The PCR-amplified constitutively expressed *Brucella* translation initiation factor IF-1 gene (BMEI1671) [39] was used as positive and homogeneity controls, and PCR-amplified *Arabidopsis thaliana* gene (*porB*, *protochlorophyllide oxidoreductase B*) was used as negative control. The microarray had a total of 7.680 spots and represented over 96.4% of the complete coding sequences assigned to *Brucella*.

### Probe preparation and microarray hybridization

The aRNA from *Brucella* wild-type and *bvrR* mutant cells were labeled with Cy3 fluorescent dye (Amersham Bioscience). Previous to the hybridization process, the microarray slides were blocked by washing with 5x SSC, 0.1% (w/v) SDS, and 1% (w/v) bovine serum albumin, pre-heated to 42°C. After 45 min at 42°C, the microarray slides were washed with water at room temperature, and then with isopropanol. The slide was then allowed to dry. Samples containing 10 µg of Cy3 labeled aRNA were dissolved in 25 µL of a solution containing 50% (v/v) deionized formamide, 5x SSC, and 0.2% (w/v) SDS, pre-heated to 42°C. After 2 min at 95°C to denature the aRNA, the solution was applied to the microarray slide, covered with a 24×60 mm cover glass, and incubated into a hybridization chamber at 42°C for 18 h. After removing the cover glass, the microarray was washed twice with 1x SSC, 0.2% (w/v) SDS at 42°C, and then successively with 0.2x SSC, 0.1% (w/v) SDS ,0.2x SSC 0.05x SSC and water at room temperature.

### Data acquisition and gene expression analysis

Fluorescent images were generated by scanning the slides using a GenePix 4100A microarray scanner (Amersham Bioscience) at 600 PMT Gain and with filter 670DF40. Spot intensity was determined using the software packages Genepix Pro 5.0 (Axon). The raw fluorescence intensity data was adjusted for background. Six measurements per gene were made, representing three independent RNA extractions of *Brucella* cells, since each gene is present twice on each slide. Data were statistically analyzed using the free software R and Bioconductor packages (http://www.bioconductor.org/). Normalization was made by quantiles and the statistical analysis was made with the t-test with FDR control (p<0.01). Microarray data have been deposited in the EMBL-EBI ArrayExpress repository (http://www.ebi.ac.uk/microarray-as/aer/#ae-main[0]) with the accession number E-MEXP-2564.

### Quantitative real-time PCR (RT-PCR)

Validation of microarray results was made by RT-PCR. Briefly, total RNA were reverse transcribed into cDNA using random oligonucleotide hexamers and SuperScript III RT (Invitrogen) according to manufacturer's protocol. Then, one µL of the resulting cDNA was used in quantitative real-time PCR reactions using Power SYBR® Green PCR Master Mix (Applied Biosystems) and a 7500 Real Time PCR System (Applied Biosystems). Primers (Table S2) were designed using Primer Express 3.0 software (Applied Biosystems). To confirm the lack of DNA contamination, reactions without reverse transcriptase were performed. Dissociation curve analysis was performed for verification of product homogeneity. Threshold fluorescence was established within the geometric phase of the exponential amplification and the cycle of threshold (Ct) was determined for each reaction. The reactions were made by triplicate from at least two independent cultures. Data were normalized by the 2^-ΔΔCt^ method [9] using the IF-1 housekeeping gene of *Brucella* [39] as reference 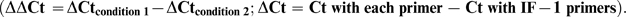


### Cell culture, infection and isolation of *Brucella* RNA from cells

Baby hamster kidney (BHK-21) cells were cultured at 37°C with 5% CO_2_ atmosphere in GMEM (Glasgow's modified Eagle's medium, Gibco) supplemented with 10% Tryptose Phosphate Broth (Sigma Aldrich) and 5% fetal calf serum (FCS, Hyclone), and seeded 24 h before infection on 55 cm^2^ culture dishes (1.5×10^6^ cells per dish). Infections were performed at a multiplicity of infection of 400∶1 by centrifuging bacteria (*B. abortus* 2308) onto BHK-21 cells at 400 g for 10 min at 4°C, and then by incubating cells for 1 h at 37°C under a 5% CO2 atmosphere. Cells were extensively washed with GMEM to remove extracellular bacteria and were incubated for an additional hour in the same medium with 50 µg/mL gentamycin to kill extracellular bacteria. Thereafter, the antibiotic concentration was decreased to 10 µg/mL. *Brucella* intracellular survival was monitored by fluorescence microscopy as describe previously [40].

To obtain RNA from *Brucella*-infected cells, a total of twelve 55 cm^2^ culture dishes were used. Cells were washed four times with 10 mL of PBS, and absence of extracellular bacteria was tested by plating onto TSA plates. BHK-21 infected cells were lysed by adding 1% Triton (Sigma Aldrich) at room temperature for 5 min, and collected by centrifugation. Cell RNA/DNA was enzymatically digested by incubation with DNase (20 U, Ambion) and RNase (20 U, Riboshredder, Epicentre) for 30 min at 37°C. Bacteria were pelleted at 8.000 g for 2 min. To lyse the remaining bacteria and isolated intracellular *Brucella* RNA, the same method was used, as described above.

## Supporting Information

Table S1 Candidate BvrS/BvR-regulated genes identified by microarray analysis. This table is a complete list of differentially expressed genes in the *Brucella* bvrR mutant versus the wild type strain.
(0.16 MB DOC)

Table S2 PCR primers used in this study.
(0.07 MB DOC)
