# Supplementary material for: Transcriptome Analysis of the Brucella abortus BvrR/BvrS Two-Component Regulatory System
Source: PLoS One. 2010 Apr 21;5(4):e10216. doi: 10.1371/journal.pone.0010216 (PMC2858072; doi:10.1371/journal.pone.0010216)
Supplement: Table S1 — Candidate BvrS/BvR-regulated genes identified by microarray analysis. This table is a complete list of differentially expressed genes in the Brucella bvrR mutant versus the wild type strain. (0.16 MB DOC) [file pone.0010216.s001.doc]

| **Up regulated** |  |
| --- | --- |
| ***B. abortus* ORF** | **Gene name/Predicted function** |
| [BAB1_0017](http://www.genome.jp/dbget-bin/www_bget?bmf:BAB1_0017) | Hydroxymethylglutaryl-CoA lyase |
| [BAB1_0020](http://www.genome.jp/dbget-bin/www_bget?bmf+BAB1_0020) | Ivd, isovaleryl-CoA dehydrogenase |
| [BAB1_0067](http://www.genome.jp/dbget-bin/www_bget?bmf:BAB1_0067) | Hypothetical cytosolic protein |
| [BAB1_0186](http://www.genome.jp/dbget-bin/www_bget?bmf:BAB1_0186) | Hypothetical protein |
| [BAB1_0216](http://www.genome.jp/dbget-bin/www_bget?bmf:BAB1_0216) | ThiG, thiazole synthase |
| [BAB1_0253](http://www.genome.jp/dbget-bin/www_bget?bmf:BAB1_0253) | Hypothetical protein |
| [BAB1_0260](http://www.genome.jp/dbget-bin/www_bget?bmf:BAB1_0260) | FlgJ, flagellar protein |
| [BAB1_0320](http://www.genome.jp/dbget-bin/www_bget?bmf:BAB1_0320) | Acyl-CoA synthetase |
| [BAB1_0338](http://www.genome.jp/dbget-bin/www_bget?bmf:BAB1_0338) | Hypothetical protein |
| [BAB1_0372](http://www.genome.jp/dbget-bin/www_bget?bmf:BAB1_0372) | Mannitol transporter |
| [BAB1_0373](http://www.genome.jp/dbget-bin/www_bget?bmf:BAB1_0373) | Extracellular solute-binding protein |
| [BAB1_0421](http://www.genome.jp/dbget-bin/www_bget?bmf:BAB1_0421) | Hypothetical protein |
| [BAB1_0500](http://www.genome.jp/dbget-bin/www_bget?bmf:BAB1_0500) | SurF1 family protein |
| [BAB1_0505](http://www.genome.jp/dbget-bin/www_bget?bmf:BAB1_0505) | S7, ribosomal protein |
| [BAB1_0568](http://www.genome.jp/dbget-bin/www_bget?bmf:BAB1_0568) | IolE, sugar phosphate isomerase/epimerase |
| [BAB1_0579](http://www.genome.jp/dbget-bin/www_bget?bmf:BAB1_0579) | L-asparaginase II |
| [BAB1_0581](http://www.genome.jp/dbget-bin/www_bget?bmf:BAB1_0581) | Death-on-curing family protein |
| [BAB1_0597](http://www.genome.jp/dbget-bin/www_bget?bmf:BAB1_0597) | Hypothetical cytosolic protein |
| [BAB1_0604](http://www.genome.jp/dbget-bin/www_bget?bmf:BAB1_0604) | Hypothetical cytosolic protein |
| [BAB1_0778](http://www.genome.jp/dbget-bin/www_bget?bmf:BAB1_0778) | Arginyltransferase |
| [BAB1_0885](http://www.genome.jp/dbget-bin/www_bget?bmf:BAB1_0885) | CobS, cobalamin synthase |
| [BAB1_0977](http://www.genome.jp/dbget-bin/www_bget?bmf:BAB1_0977) | FumB, fumarate hydratase, class I |
| [BAB1_1022](http://www.genome.jp/dbget-bin/www_bget?bmf:BAB1_1022) | Hypothetical protein |
| [BAB1_1043](http://www.genome.jp/dbget-bin/www_bget?bmf:BAB1_1043) | Hypothetical membrane spanning protein |
| [BAB1_1357](http://www.genome.jp/dbget-bin/www_bget?bmf:BAB1_1357) | Short chain dehydrogenase |
| [BAB1_1366](http://www.genome.jp/dbget-bin/www_bget?bmf:BAB1_1366) | FhuD, ABC transporter |
| [BAB1_1368](http://www.genome.jp/dbget-bin/www_bget?bmf:BAB1_1368) | YadH, ABC transporter |
| [BAB1_1397](http://www.genome.jp/dbget-bin/www_bget?bmf:BAB1_1397) | Aminotransferase, class I |
| [BAB1_1573](http://www.genome.jp/dbget-bin/www_bget?bmf:BAB1_1573) | MoxR, chaperonin protein |
| [BAB1_1601 BAB1_1602](http://www.genome.jp/dbget-bin/www_bget?bmf+BAB1_1601) | OppA, peptide ABC transporter |
| [BAB1_1620](http://www.genome.jp/dbget-bin/www_bget?bmf:BAB1_1620) | Glycosyl transferase, family 25 |
| [BAB1_1624](http://www.genome.jp/dbget-bin/www_bget?bmf:BAB1_1624) | PotC, ABC transporter |
| [BAB1_1662](http://www.genome.jp/dbget-bin/www_bget?bmf:BAB1_1662) | Glutamyl-Q tRNA(Asp) synthetase |
| [BAB1_1821](http://www.genome.jp/dbget-bin/www_bget?bmf:BAB1_1821) | HtpX, heat shock protein |
| [BAB1_1832](http://www.genome.jp/dbget-bin/www_bget?bmf:BAB1_1832) | RpsP, 30S ribosomal protein S16 |
| [BAB1_1868](http://www.genome.jp/dbget-bin/www_bget?bmf:BAB1_1868) | ClpB, chaperonin |
| [BAB1_1879](http://www.genome.jp/dbget-bin/www_bget?bmf:BAB1_1879) | GrxC, glutaredoxin 3 |
| [BAB1_2043](http://www.genome.jp/dbget-bin/www_bget?bmf:BAB1_2043) | FabG,3-ketoacyl-(acyl-carrier-protein) reductase |
| [BAB1_2044](http://www.genome.jp/dbget-bin/www_bget?bmf:BAB1_2044) | Oxidoreductase |
| [BAB1_2062](http://www.genome.jp/dbget-bin/www_bget?bmf:BAB1_2062) | GidA, tRNA modification enzyme |
| [BAB1_2064](http://www.genome.jp/dbget-bin/www_bget?bmf:BAB1_2064) | Thymidylate kinase |
| [BAB1_2091](http://www.genome.jp/dbget-bin/www_bget?bmf+BAB1_2091) | PckA, phosphoenolpyruvate carboxykinase |
| [BAB2_0032](http://www.genome.jp/dbget-bin/www_bget?bmf:BAB2_0032) | Branched-chain a-keto acid dehydrogenase subunit E3 |
| [BAB2_0037](http://www.genome.jp/dbget-bin/www_bget?bmf:BAB2_0037) | Hypothetical protein |
| [BAB2_0124 BAB2_0125](http://www.genome.jp/dbget-bin/www_bget?bmf+BAB2_0125) | FliM, flagellar motor switch protein |
| [BAB2_0130](http://www.genome.jp/dbget-bin/www_bget?bmf:BAB2_0130) | Hypothetical protein |
| [BAB2_0351](http://www.genome.jp/dbget-bin/www_bget?bmf:BAB2_0351) | osmotically inducible protein C |
| [BAB2_0441](http://www.genome.jp/dbget-bin/www_bget?bmf:BAB2_0441) | Hypothetical protein |
| [BAB2_0443](http://www.genome.jp/dbget-bin/www_bget?bmf:BAB2_0443) | Acetyl-CoA acetyltransferase |
| [BAB2_0511](http://www.genome.jp/dbget-bin/www_bget?bmf:BAB2_0511) | Oxidoreductase |
| [BAB2_0552](http://www.genome.jp/dbget-bin/www_bget?bmf:BAB2_0552) | Hypothetical protein |
| [BAB2_0572](http://www.genome.jp/dbget-bin/www_bget?bmf:BAB2_0572) | Aminotransferase |
| [BAB2_0597](http://www.genome.jp/dbget-bin/www_bget?bmf:BAB2_0597) | PcaC, carboxymuconolactone decarboxylase |
| [BAB2_0604](http://www.genome.jp/dbget-bin/www_bget?bmf:BAB2_0604) | PcaI, coenzyme A transferase 1 |
| [BAB2_0606](http://www.genome.jp/dbget-bin/www_bget?bmf:BAB2_0606) | Acetyl-coa acetyltransferase |
| [BAB2_0627](http://www.genome.jp/dbget-bin/www_bget?bmf:BAB2_0627) | Hypothetical protein |
| [BAB2_0660](http://www.genome.jp/dbget-bin/www_bget?bmf:BAB2_0660) | Hypothetical cytosolic protein |
| [BAB2_0690](http://www.genome.jp/dbget-bin/www_bget?bmf:BAB2_0690) | Endoglucanase family protein |
| [BAB2_0697](http://www.genome.jp/dbget-bin/www_bget?bmf:BAB2_0697) | Hypothetical protein |
| [BAB2_0712](http://www.genome.jp/dbget-bin/www_bget?bmf:BAB2_0712) | LpdA-3, dihydrolipoamide dehydrogenase |
| [BAB2_0716](http://www.genome.jp/dbget-bin/www_bget?bmf:BAB2_0716) | Hypothetical protein |
| [BAB2_0737](http://www.genome.jp/dbget-bin/www_bget?bmf:BAB2_0737) | Putative monovalent cation/H+ antiporter subunit C |
| [BAB2_0749](http://www.genome.jp/dbget-bin/www_bget?bmf+BAB2_0749) | Hypothetical protein |
| [BAB2_0794 BAB2_0795](http://www.genome.jp/dbget-bin/www_bget?bmf+BAB2_0794) | Mfs family transporter |
| [BAB2_0803](http://www.genome.jp/dbget-bin/www_bget?bmf+BAB2_0803) | Fucose synthetase family protein |
| [BAB2_0863](http://www.genome.jp/dbget-bin/www_bget?bmf:BAB2_0863) | Glutaminase |
| [BAB2_0892](http://www.genome.jp/dbget-bin/www_bget?bmf+BAB2_0892) | Hypothetical protein |
| [BAB2_0917](http://www.genome.jp/dbget-bin/www_bget?bmf:BAB2_0917) | SsuB,ABC transporter |
| [BAB2_0928](http://www.genome.jp/dbget-bin/www_bget?bmf:BAB2_0928) | NosZ, Nitrous-oxide reductase |
| BAB2_0951 | Hypothetical protein |
| BAB2_0955 | NorC, nitric-oxide reductase |
| [BAB2_0999](http://www.genome.jp/dbget-bin/www_bget?bmf:BAB2_0999) | Selenoprotein W-related |
| [BAB2_1000](http://www.genome.jp/dbget-bin/www_bget?bmf:BAB2_1000) | Hypothetical cytosolic protein |
| [BAB2_1051](http://www.genome.jp/dbget-bin/www_bget?bmf:BAB2_1051) | DppC, ABC transporter |
| [BAB2_1085](http://www.genome.jp/dbget-bin/www_bget?bmf:BAB2_1085) | Hypothetical cytosolic protein |
| [BAB2_1103](http://www.genome.jp/dbget-bin/www_bget?bmf:BAB2_1103) | MotB, flagellar motor protein |
| [BAB2_1107](http://www.genome.jp/dbget-bin/www_bget?bmf:BAB2_1107) | Cell wall surface protein |
| [BAB2_1114](http://www.genome.jp/dbget-bin/www_bget?bmf:BAB2_1114) | Aldehyde dehydrogenase |
| [BAB2_1127](http://www.genome.jp/dbget-bin/www_bget?bmf:BAB2_1127) | Hypothetical protein |
| [BAB2_1130](http://www.genome.jp/dbget-bin/www_bget?bmf:BAB2_1130) | Aldehyde dehydrogenase |
| [BAB2_1136](http://www.genome.jp/dbget-bin/www_bget?bmf:BAB2_1136) | Hypothetical protein |
| [BAB2_1139](http://www.genome.jp/dbget-bin/www_bget?bmf+BAB2_1139) | Sugar ABC transporter |
| [BAB2_1152](http://www.genome.jp/dbget-bin/www_bget?bmf:BAB2_1152) | Transcriptional regulator, AraC family |

| **Down regulated** |  |
| --- | --- |
| ***B. abortus* ORF** | **Gene name/Predicted function** |
| [BAB1_0041](http://www.genome.jp/dbget-bin/www_bget?bmf:BAB1_0041) | CyoC, cytochrome c oxidase,subunit III |
| [BAB1_0097](http://www.genome.jp/dbget-bin/www_bget?bmf:BAB1_0097) | Hypothetical protein |
| [BAB1_0115](http://www.genome.jp/dbget-bin/www_bget?bmf:BAB1_0115) | Omp25d, outer membrane protein |
| [BAB1_0236](http://www.genome.jp/dbget-bin/www_bget?bmf:BAB1_0236) | amydohydrolase 2 |
| [BAB1_0237](http://www.genome.jp/dbget-bin/www_bget?bmf:BAB1_0237) | IclR, transcriptional regulator |
| [BAB1_0238](http://www.genome.jp/dbget-bin/www_bget?bmf:BAB1_0238) | Sugar ABC transporter |
| [BAB1_0239](http://www.genome.jp/dbget-bin/www_bget?bmf:BAB1_0239) | MalF, maltose ABC transporter permease sugar |
| [BAB1_0240](http://www.genome.jp/dbget-bin/www_bget?bmf:BAB1_0240) | MalG, sugar ABC transporter |
| [BAB1_0241](http://www.genome.jp/dbget-bin/www_bget?bmf:BAB1_0241) | MalK, sugar ABC transporter |
| [BAB1_0242](http://www.genome.jp/dbget-bin/www_bget?bmf:BAB1_0242) | Mandelate racemase |
| [BAB1_0243](http://www.genome.jp/dbget-bin/www_bget?bmf:BAB1_0243) | Hypothetical protein |
| [BAB1_0246](http://www.genome.jp/dbget-bin/www_bget?bmf:BAB1_0246) | UcpA, oxidoreductase |
| [BAB1_0248](http://www.genome.jp/dbget-bin/www_bget?bmf:BAB1_0248) | Mandelate racemase |
| [BAB1_0340](http://www.genome.jp/dbget-bin/www_bget?bmf:BAB1_0340) | MATE efflux family protein |
| [BAB1_0358](http://www.genome.jp/dbget-bin/www_bget?bmf:BAB1_0358) | Lipoprotein |
| [BAB1_0383](http://www.genome.jp/dbget-bin/www_bget?bmf:BAB1_0383) | Guanine deaminase |
| [BAB1_0453](http://www.genome.jp/dbget-bin/www_bget?bmf:BAB1_0453) | Hypothetical protein |
| [BAB1_0476](http://www.genome.jp/dbget-bin/www_bget?bmf:BAB1_0476) | Cfa, methyltransferase |
| [BAB1_0504](http://www.genome.jp/dbget-bin/www_bget?bmf:BAB1_0504) | Thiol peroxidase |
| [BAB1_0526](http://www.genome.jp/dbget-bin/www_bget?bmf:BAB1_0526) | Polysaccharide deacetylase |
| [BAB1_0589](http://www.genome.jp/dbget-bin/www_bget?bmf:BAB1_0589) | Lipoprotein |
| [BAB1_0666](http://www.genome.jp/dbget-bin/www_bget?bmf:BAB1_0666) | DapA, dihydrodipicolinate synthase |
| [BAB1_0703](http://www.genome.jp/dbget-bin/www_bget?bmf:BAB1_0703) | Peptidase M23/M37 |
| [BAB1_0716](http://www.genome.jp/dbget-bin/www_bget?bmf:BAB1_0716) | Glycoprotein |
| [BAB1_0722](http://www.genome.jp/dbget-bin/www_bget?bmf:BAB1_0722) | Omp25a, outer membrane protein |
| [BAB1_0724](http://www.genome.jp/dbget-bin/www_bget?bmf:BAB1_0724) | Ribonuclease T2 family protein |
| [BAB1_0805](http://www.genome.jp/dbget-bin/www_bget?bmf:BAB1_0805) | ATPase |
| [BAB1_0814](http://www.genome.jp/dbget-bin/www_bget?bmf+BAB1_0814) | Hypothetical membrane spanning protein |
| [BAB1_0872](http://www.genome.jp/dbget-bin/www_bget?bmf:BAB1_0872) | FabF, acyltransferase |
| [BAB1_0891](http://www.genome.jp/dbget-bin/www_bget?bmf:BAB1_0891) | ExoR,exopolysacchride production negative regulator |
| BAB1_0903 | TatC, sec-independent periplasmic protein |
| [BAB1_1461](http://www.genome.jp/dbget-bin/www_bget?bmf:BAB1_1461) | Transglycosylase SLT domain protein |
| [BAB1_1717](http://www.genome.jp/dbget-bin/www_bget?bmf:BAB1_1717) | Hypothetical protein |
| [BAB1_2017](http://www.genome.jp/dbget-bin/www_bget?bmf:BAB1_2017) | Hypothetical protein |
| [BAB1_2093](http://www.genome.jp/dbget-bin/www_bget?bmf:BAB1_2093) | BvrS, sensor protein |
| [BAB1_2094](http://www.genome.jp/dbget-bin/www_bget?bmf:BAB1_2094) | HPr-K |
| [BAB1_2147](http://www.genome.jp/dbget-bin/www_bget?bmf:BAB1_2147) | Lipoprotein |
| [BAB2_0033](http://www.genome.jp/dbget-bin/www_bget?bmf:BAB2_0033) | Shikimate dehydrogenase family protein |
| [BAB2_0118](http://www.genome.jp/dbget-bin/www_bget?bmf:BAB2_0118) | vjbR,transcriptional regulator, LuxR family |
| [BAB2_0762](http://www.genome.jp/dbget-bin/www_bget?bmf:BAB2_0762) | OmpR,transcriptional regulatory protein |
| BAB2_0817 | AppD, peptide ABC transporter |
| [BAB2_0943](http://www.genome.jp/dbget-bin/www_bget?bmf:BAB2_0943) | NirK, Nitrite reductase |
| [BAB2_1120](http://www.genome.jp/dbget-bin/www_bget?bmf:BAB2_1120) | HpcD, 5-carboxymethyl-2-hydroxymuconate isomerase |
| [BAB2_1143](http://www.genome.jp/dbget-bin/www_bget?bmf:BAB2_1143) | UgpC, Sugar ABC transporter |
