# Supplementary material for: Transcriptome Analysis of the Brucella abortus BvrR/BvrS Two-Component Regulatory System
Source: PLoS One. 2010 Apr 21;5(4):e10216. doi: 10.1371/journal.pone.0010216 (PMC2858072; doi:10.1371/journal.pone.0010216)
Supplement: Table S2 — PCR primers used in this study. (0.07 MB DOC) [file pone.0010216.s002.doc]

| *B. abortus* ORF | Forward Primer (5'-3') | Reverse Primer (5'-3') |
| --- | --- | --- |
| BAB1_0017 | TCGGTTCTCGTGCCCAATA | CGCTCGATACTTTCCGCAAT |
| BAB1_0115 | GGCGGTGTTGCCTTTGG | TCCAGACGCAGAAGAACATTGT |
| BAB1_0237 | GGCTCGGTGGTGGTCATC | GGGCGGCAATCATCATTTC |
| BAB1_0239 | TGCGGTGGTGCTCTTTCC | CAGAACACCCAGTCACTCAGGAT |
| BAB1_0246 | GCGCTGGTGGCTGAAATC | GACATTCAGGTTGACGGCAAA |
| BAB1_0260 | CCAAGGTTTCAAGGGCAGTTT | CGTGTCCGCATTTTTCTTGTC |
| BAB1_0282 | ATGGCGAAAGAAGAAGTCCT | ACCAGAACCTTGTCACCGGC |
| BAB1_0320 | TCCGATTACCTTTACCGCAGTT | ACGACGAGCGCCTTTGC |
| BAB1_0358 | GGTGTGCTGGTGCCTATCG | TTTACCTCGTTCGCCAGAGAA |
| BAB1_0476 | CGATGCCGATGGTTCAAAA | TCTCGCCATCCATGAAACATT |
| BAB1_0526 | GGCAAGACAGATCATCGCATT | TCGGGATGAGCCTTGAGAAC |
| BAB1_0568 | GGTGGAAACAGCCTTTGATGA | GCTCCAGAAACATCTCAAAAGTCA |
| BAB1_0589 | CAAATGTATGCTGCGGTAGAAGA | GCACCAGATAGCAGAAGCGATT |
| BAB1_0666 | GCCGTGTGCCAGTGATTG | CACGCTGATTCGGCTTGTT |
| BAB1_0716 | GGGCAGTGGCGTGATTG | AGGCGATGTTTTCACCAGTCA |
| BAB1_0722 | TCTGCGACCGCTTTTGCT | TTCCAGCCATAGCCAAGGTAA |
| BAB1_0805 | TGGATGCGCTGGAAACG | GCAGAAATAGCCACGGCATT |
| BAB1_0872 | CCCATCGTCGCCATCAG | GAAAATCCACCGTACCGGAAA |
| BAB1_0891 | TGGTCCTTACCTTGGCACTTG | CGTCCTTATGCCCGTTCTTG |
| BAB1_0977 | CACCCCTTATCGCAAACTGACT | GCGCAGCAGATGGTTGATATC |
| BAB1_1366 | TCTGCCGCTGGAGAAACTG | GCCCGCGCCTGCTT |
| BAB1_1368 | GAGCCTTGGCATTTTTTTGG | GCAAGCATGATCGTCTGAATGA |
| BAB1_1397 | GCACATGCCGACGAACTG | TGGAGAACGAGCCGCAATAG |
| BAB1_1573 | AGCATCGGCACCGTAATCTT | CGCTGGCCGGAAAGG |
| BAB1_1620 | GAGCGTGTGGAAGCCGTAAA | CGATGAGAAAGAAAACAGCCAAT |
| BAB1_1624 | CGCTGCTTCTGCTCTTCGT | CGCCGCTTCTTCCAGTGA |
| BAB1_1821 | CCGAATGAAGAAGCCGAACA | ACCCATTTCAGCAGCCATCT |
| BAB1_2043 | TTGCCCTGAAGCCTGAAAAT | CGATGGAGCGTGGAAAGC |
| BAB1_2091 | GGCAGGCACGGAAAAGG | GACCAGCCAGCAATCAACCT |
| BAB1_2093 | GCGTGTTCGGCGTTGTG | CTGACGCTCGGAAAAATCG |
| BAB1_2094 | AGGGCGACAGGTTGATTGC | AGCCTCGTCGCCATTCAC |
| BAB1_2147 | AGACCAGCTCCAGCGATCAG | GGAGCGGTTGGATTCAAAGA |
| BAB2_0032 | GTCGTATTGCCTGCCCTTTC | CCATCCACTTCGGCTTCAA |
| BAB2_0118 | CGATACTTTGGCGCTTCTAACC | AGGTAGCAGGCAGCGTCATAA |
| BAB2_0124-25 | CGCTGGAGCATAACCGTAAAA | AGCCGGGCGCAGAAA |
| BAB2_0130 | GCAAGGGTGACCGCTATCTG | TGCGCTCAGCCTTGTTTG |
| BAB2_0351 | GGCGGCGAAAGAAAAGATT | GTCGATGCCAGGAAGAGCAA |
| BAB2_0443 | CCGCAGGGCGTTTCC | GGCCGTTCTGGTCCTTGAT |
| BAB2_0712 | CGATGGAACGCGATGATG | TTCCACCGCCTGAGCAA |
| BAB2_0762 | AAAAGCGGCATAGAAATCTGTCA | CGAAGGGCTTGGCGATATAGT |
| BAB2_0863 | GCTTTAGGCGATTGGGCTTA | GCCATTGCTTTCACACTATTTCC |
| BAB2_0928 | CGTGTACCGATCCCGAATG | GCCCTGATAGTCGCAATCCA |
| BAB2_0943 | ATTATGTCGGCGAGCAGGAT | CACCCACCGCACCATTG |
| BAB2_0955 | CCTGAAAGTGCCCGTGAAAC | GCTCGTCCATTCCAGGAAATC |
| BAB2_1103 | AGGCTGAAACGCAGGAAGAA | ACGGGAACCACGGTAACATC |
| BAB2_1127 | CGCTTGGCACGGACCTT | CCCCCCGGCCCATAG |
| BAB2_1130 | CGTGCGGGACAATGGAA | CCGTGGGCGCGATCT |
| BAB2_1152 | CGCAAGCCTTTGGTTTTCAT | GTGGTTTCCTGTGCTTCATCCT |
